# Uterine artery chemoembolization compared with uterine artery embolization combined with prior to hysteroscopy and curettage in the treatment of cesarean scar pregnancy

**DOI:** 10.4314/ahs.v25i1.43

**Published:** 2025-03

**Authors:** Liangping Wang, Weiye Cheng, Yiqi Zhao, Hanbo Liu

**Affiliations:** 1 Center for Reproductive Medicine, Department of Reproductive Endocrinology, Zhejiang Provincial People's Hospital (Affiliated People's Hospital, Hangzhou Medical College), Hangzhou, China; 2 Cancer Center, Department of Interventional Medicine, Zhejiang Provincial People's Hospital (Affiliated People's Hospital, Hangzhou Medical College), Hangzhou, China

**Keywords:** Uterine artery embolization, Chemoembolization, Methotrexate, Hysteroscopic surgery, Cesarean scar pregnancy

## Abstract

**Background:**

To compare evaluate the clinical effectiveness and safety efficacy of uterine artery chemoembolization (UACE) and versus uterine artery embolization (UAE) prior to combined with hysteroscopy and curettage in the treatment of cesarean scar pregnancy (CSP)

**Methodsology:**

A total The clinical data of 84 patients with CSP who underwent UACE or U AE combined with hysteroscopy and-guied uterine curettage were enrolled in the retrospective cohort study from August 2016 to April 2021 were retrospectively collected. They were divided into UACE group (n = 39) and UAE group (n = 45) according to different surgical methods. The clinical characteristics general data, operat treatment success rate, ion, postoperative outcome conditions, and re-pregnancy productive outcomes of the two groups were observed and compared and analyzed between the two groups.

**Results:**

The overall success rate of 84 patients was 96.43%, the complication rate was 19.05%, and the serious complication rate was 1.19%. There was no significant difference between the two groups in the success rate of operation, complication rate, serious complication rate, UAE operativeon time, hysteroscopic operation time, intraoperative bleeding volume, duration of postoperative hospital stay, β-human chorionic gonadotropin (β-hCG) reduction decrease on postoperative day 1, and re-pregnancy productive outcome (P > 0.05). The time of menstruation recoveryto resumption of menses was shorter in the UACE group than in the UAE group (P < 0.05).

**Conclusion:**

UACE was as effective and safe as UAE in the treatment of CSPor UAE combined with hysteroscopy is safe and effective in the treatment of CSP, and the time of menstruation recovery was shorter. UACE reduced the time to resumption of menses compared with UAE, with a similar both of which had similar re-pregnancy productive outcomes.

## Introduction

Cesarean scar pregnancy (CSP) after cesarean section refers to the implantation of the fertilized egg at the uterine incision scar of the previous cesarean section. CSP is a long-term serious complication after cesarean section. If CSP is not treated in time, placenta praevia, placenta implantation, and uterine rupture can occur, leading to uncontrollable haemorrhage and, in severe cases, hysterectomy or even life-threatening injuries. According to statistics, The estimated incidence of CSP range from its incidence is 1:1800 to 1:2656 of overall pregnancies216, accounting for 6.1%^1^ of ectopic pregnancies in women with a history of cesarean section. Based on the depth of the implantation, CSP can be categorized as either endogenic type or exogenic type^1^. The cesarean section rate is high in China, and the occurrence of CSP is closely related to the history of cesarean section. With the adjustment of fertility policy in China, the diagnosis and treatment of CSP has have received more and more attention. The main treatment options for CSP include medical treatment by methotrexate (MTX), uterine curettagesystemic and local chemotherapy of the pregnancy tissue, hysteroscopy, uterine artery embolization (UAE), uterine artery chemoembolization (UACE), laparoscopyic surgery, hysteroscopic surgery, high high-intensity focused ultrasound, hysterectomy, etc.^2^. However, there is no clinical consensus on the optimal treatment for CSP^3^. In this study, we retrospectively analyzed the clinical data of 84 patients who underwent UACE or UAE prior to combined with hysteroscopy and-guided uterine curettage for CSP in a single center to explore whether UACE is as evaluate the effective and safe as efficacy and safety of UACE and UAE in the treatment of CSP.

### Material Patients and Methods Patients

This retrospective study was conducted in the Zhejiang Provincial People's Hospital, Hangzhou, China. 84 CSP patients with CSP undergoing UACE or UAE prior to combined with hysteroscopyic and curettagesurgery were enrolled admitted to Zhejiang Provincial People's Hospital from August 2016 to April 2021. Inclusion criteria were set as follows: 1. Patients with a Ddiagnosis of CSP according to medical historyin combination with history of previous caesarean section, elevated serum beta-hCG level, and imaging examinations(ultrasound examination or MRI); 2. Intraoperative confirmation and postoperative pathology (suggestive of chorionic tissue implantation in the myometrium or visible chorionic tissue) confirmed the diagnosis of CSP; 3. The patients had complete clinical data were complete. The patients who received sex hormone therapy, or chemotherapy within 6 months before the treatment cervical pregnancy, trophoblastic disease, and laparoscopic scar repair were excluded. At the time ofOn admission, 50 patients had irregular vaginal bleeding, 11 had lower abdominal pain, 1 had nausea and vomiting, and the rest had no significant symptoms. 3 patients had a previous history of CSP and were treated by curettage. The serum β-hCG was (43037.92±39545.09) mIU/ml at admission and the maximum diameter of the gestational sac was (29.21±14.10) mm on ultrasound. All patients were divided into UACE group and UAE group according to the operation with or without the presence or absence of intraoperative arterial infusion of methotrexate (MTX) administration. The Hysteroscopyically and curettage were performed guided hysterectomy under general anaesthesia within 24-48 h after UACE or UAE.

## Methods

The Routine examinations were perfected preoperatively in all patients. (1) UAE procedure: The operation was performed under thea Allura FD20 (Philips Corporation, Belgium) flat panel digital subtraction angiography (DSA) system. After a successful puncture of the right femoral artery using a modified Seldinger technique, a 5F catheter sheath was placed. A 5F pigtail catheter (Cordis, USA) was fed through the sheath and placed in the distal abdominal aorta to visualize the pelvic vessels. 5F Yashiro catheter (Terumo, Japan) was selectively cannulated into the contralateral common iliac artery and internal iliac artery for imaging; a coaxial microcatheter technique was used. A 2.7F Progreat microcatheter (Terumo, Japan) was superselected to the distal uterine artery and a total of 4 ml of contrast was contrasted at a flow rate of 1.5 ml/s for visualization. MTX was slowly injected into the uterine artery via the catheter (UACE group used) and then slowly embolized with an appropriate amount of 500-1000 µm gelatin sponge pellets (Allegra Pharmaceutical Technology, Hangzhou, China). The right uterine artery was embolized in the same way. Internal iliac artery angiography was repeated and superselective embolization was performed if there were collateral vessels. The endpoint of embolization was that the uterine artery was largely stagnant and no branches were visible. The Hysteroscopic surgery: Hhysteroscopyically and curettageguided hysterectomy followed by endovascular treatment were performed under general anaesthesia within 24-48 h after UAE. Clamp scraping was performed under direct hysteroscopic view and the scrapings were sent for pathological examination after the procedure.

### Statistical indicators

The demographic and clinical characteristics of the two groups were analyzed. of patients were compared in terms of preoperative general information, number of pregnancies and deliveries, preoperative CSP typing, serum β-hCG levels, etc.; Tthe operationsurgical and postoperative outcomesconditions of the two groups, including the operative timeduration of surgery, intraoperative bleeding volume, duration of postoperative hospital stay, surgery-related complications (embolism syndromes, such as fever, abdominal pain, nausea and vomiting, thrombosis, ectopic embolism, etc.), serious complications (hysterectomy, abdominal surgerycaesarean section, bleeding volume > 1000 ml, blood transfusion), and re-pregnancy outcome, etc.were collected and analyzed. Patients were followed up by telephone after discharge from the hospital.in outpatient clinics or by telephone for biochemical indicators and fertility.

### Statistical analysis

Statistical analysis was performed using Statistical Product and Service Solutions (SPSS), Version 25.0 software (IBM Corp., Armonk, NY, USA). Continuous variables Measurement data were describedexpressed as (x ± s). The Independent sample t-test was used for inter-group comparison. The categorical variables Enumeration data werexpressed as frequency (percentage). The χ2 or Fisher exact test was used for inter-group comparison. A value of P < 0.05 was considered statistically significant.

## Results

Baseline demographics and clinical characteristics of the patientsClinical data comparison As shown in Table 1, In the UACE group (39 patients), the mean patient age was (35.82 ± 6.00); in the UAE group (45 patients), the mean patient age was (36.96 ± 5.10). There wereas no significant differences in age, gravidity, number of cesarean sections, last cesarean section time, symptom of vaginal bleeding, and gestational age duration of menopause between the two groups (P > 0.05). Ultrasonography and laboratory tests were perfected on admission, and there were no significant differences in maximum gestational sac diameter, presence or absence of blastema, myometrial thickness, type of C SP classification, and serum β-hCG levels between the two groups (P > 0.05) (see Table 1).

**Table 1:** Baseline demographics and clinical characteristics of the patients

I	UACE group (n = 39)	UAE group (n = 45)	t/χ^2^	P value
Age, y	35.82 ± 6.00	36.96 ± 5.10	-0.937	0.351
Gravidity	3.64 ± 1.65	3.58 ± 1.67	0.174	0.862
Number of cesarean section	1.59 ± 0.50	1.56 ± 0.59	0.286	0.776
Time of last cesarean section, y	5.56 ± 4.38	6.47 ± 4.66	-0.910	0.365
Symptom of vaginal bleeding	26 (66.7)	24 (53.3)	1.542	0.214
Gestational age, d	49.31 ± 12.30	47.84 ± 9.07	0.626	0.533
Maximum gestational sac diameter, mm	30.59 ± 14.86	28.02 ± 13.47	0.831	0.409
Germ	20 (51.3)	29 (64.4)	1.489	0.222
Muscular layer thickness, mm	2.87 ± 1.33	3.26 ± 0.99	-1.531	0.130
Endogenic type	13 (33.3)	21 (46.7)	1.542	0.214
Serum β-hCG, mIU/ml	43753.56 ± 45320.82	42417.70 ± 34280.98	0.153	0.878

Values are presented as mean ± standard deviation, number (percentage).

### Surgical and postoperative conditions

The hysteroscopy and curettage were performed followed by endovascular treatment, UAE Intraoperative angiography showed tortuous and thickened uterine arteries and abundant uterine blood supply. All patients underwent hysteroscopic surgery within 24 - 48 h after UAE, and CSP was confirmed by postoperative pathology. and Tthe intraoperative bleeding volume during hysteroscopy curettage in UACE group was higher than that in UAE group (43.77 ± 102.38 ml vs. 17.78 ± 30.48 ml), and the decrease of β-hCG reduction on postoperative day 1 was lower than that in UAE group (68 ± 9.71% vs. 71.12 ± 6.60%), Howeverhowever, there was no statistically significant difference (P > 0.05). There were no significant differences in UAE operativeon time, hysteroscopy operation time, and duration of postoperative hospital stay between the two groups (P > 0.05). Notably during postoperative follow-up, the time of menstruation recovery to resumption of menses in the UACE group was shorter than that in the UAE group (28.95 ± 2.77 days vs. 30.62 ± 2.84 days, P < 0.05), with a and the difference was statistically significant difference. At postoperative follow-up, 5 of 84 patients gotbecame pregnant again during the follow-up period. Among them, one1 patient case occurred showed CSP again half a year later and underwent hysteroscopic uterine curettage, one1 patient occurred-case showed CSP again 2 years later and requested discharge, to return to the local hospital for treatment; three3 patientscases were intrauterine pregnancy, of which one1 patientcase underwent cesarean section at term and two2 patientscases underwent induced abortion due to personal factors (Table 2).

**Table 2:** Comparison of surgical and postoperative conditions between the two groups

I	UACE group (n = 39)	UAE group (n = 45)	t/χ^2^	P value
UAE operative time, min	50.31 ± 10.21	53.56 ± 6.79	-1.737	0.086
Curettage operative time, min	38.10 ± 24.34	32.56 ± 21.26	1.115	0.268
HIntraoperative bleeding volume, ml	43.77±102.38	17.78 ± 30.48	1.528	0.134
Postoperative hospital stay, d	3.90 ± 5.82	2.36 ± 1.32	1.619	0.113
β-hCG reduction on postoperative day 1, %	68 ± 9.71	71.12 ± 6.60	-1.743	0.085
Time of menstruation recovery, d	28.95 ± 2.77	30.62 ± 2.84	-2.724	0.008
Re-pregnancy outcome	2 (5.1)	3 (6.7)		1.000

Values are presented as mean ± standard deviation, number (percentage).

### Surgical success rate and complications

The overall success rate of surgery in 84 patients was 96.43%, and there was no significant difference in the success rate between the two groups (P > 0.05). In the UACE group, one1 patient was cured after a second curettagehysteroscopic operation fordue to postoperative residual bleeding, and one1 patient underwent a hysterectomy due toafter ineffective conservative treatment for of massive uterine bleeding under secondary to curettagehysteroscopy was ineffective. One patient in UAE group was cured by systemic MTX intramuscular injection due toa slow decline of serum β-hCG level after surgery. The overall complication rate was 19.05%, with no significant difference in complications between the two groups (12.8% vs. 24.4%, P > 0.05). Among them, twelve (12) patients presented with lower abdominal pain after the operation, three (3) patients presented with fever after the operation, with body temperature not exceeding 38.5D, all recovered after symptomatic treatment, and one (1) patient presented with right lower limb pain 3 days after the operation. Ultrasound revealed lower limb intermuscular venous thrombosis, which was cured after anticoagulant therapy. Except for one1 patientcase who had undergone aof hysterectomy in UACE group, there were no serious complications occurredsuch as uterine perforation, lower extremity deep venous thrombosis, or intraoperative bleeding in both groups (see Table 3).

**Table 3:** Surgical success rate and complications

I	UACE group (n = 39)	UAE group (n = 45)	P value	Total (n = 59)
Procedure success	37 (94.9)	44 (97.8)	0.595	57 (96.43)
Complications	5 (12.8)	11 (24.4)	0.176	16 (19.05)
Serious complications	1 (2.6)	0	0.464	1 (1.19)

Values are presented as number (percentage).

Procedure success: no additional treatment was required after hysteroscopy and curettage, Complications: embolic syndromes (fever, abdominal pain, nausea and vomiting), thrombosis, ectopic embolism, etc., Serious complications: hysterectomy, abdominal surgery, bleeding volume> 1000 ml, blood transfusion.

## Discussion

CSP can lead to uncontrollable bleeding and multiple complications in pregnant and lying-in women, which seriously threatens women's health. At present, the pathogenesis of CSP is still unclear, and early diagnosis, early termination, and early clearance are its therapeutic principles. Although there are many methods ways of to treatment for CSP, there is no consensus at home and abroad on its optimal treatment management 43-65. UAE can block the uterine artery blood supplysupplement of the uterus, cause local hypoxia in cesarean scar lesions, promote embryonic and trophoblastic necrosis, atrophy and shedding, and reduce the possibility of massive hemorrhage during and after uterine curettage.

Currently, UAE has become an important method of comprehensive treatment for CSP due tobecause of theits ability features of rapid hemostasisto stop bleeding quickly, preservatione of the the uterus, quick recovery quickly fromafter surgeryoperation, and have few complications, compared with the administration of MTX and curettage 76. Besides, UAE coulbe used combined with local MTX in the management of CSP8. Regarding the use of UAE in CSP, there are currently UAE combined with uterine clearance, UAE combined with hysteroscopic clearance, UACE combined with uterine clearance, etc. MTX is cytotoxic and is widely used for the treatment of ectopic pregnancy by inhibiting the production of intracellular tetrahydrofolate and folic acid derivatives, inhibiting trophoblastic cell growth, and causing embryonic tissue necrosis and resorption^9^,^7^. It is usually considered that UAE and MTX have a synergistic effect, which can effectively clear the gestational sac and facilitate the clearance of residual villi after surgery. In recent years, UACE has been widely reported for the treatment of CSP, but it is not yet fully clarified whether MTX arterial infusion is required during UAE surgery in patients with- treatment for CSP and how to dose it.

There were some differences between studies regarding the efficacy of UACE versus UAE^8^. It is believed that UAE and MTX have a synergistic effect, which can effectively clear the gestational sac and facilitate the clearance ofesidual villi after surgery. The study has^9^ found that compared with UAE in the treatment of CSP, UACE had similar intraoperative blood loss, shorter recovery time of postoperative serum β-hCG, lower rate of secondary treatment, but higher complication rate. It was considered that UAE should be preferred for endogenous type, and UACE should be used for exogenous type. However, it has also been shown^2^ that the reintervention rate of UAE combined with uterine curettage was 6.4%, while the reintervention rate of UAE + MTX combined with uterine curettage was 3 1.4%. Qi et al.^10^. The study retrospectively showed10 that compared among patients with UAE group, UAE + MTX group performed curettage guided by combined with ultrasound-guided uterine curettagehas a similar procedure, the success rate of surgery was higher in the UAE + MTX group than in the UAE group (77.3% vs. 89.3%, Fig. P > 0.05) and complication rates (18.2% vs. 3.6%, P > 0.05) were similar, but there was a higher uterine bleeding volume during curettage (80.25 ± 113.92 ml vs. 32.04 ± 21.41 ml, P < 0.05). Gao et al.^11^ concluded that UAE plus intra-arterial MTX and curettage significantly facilitated serum β-hCG decline on the first day and post-operative recovery, however, the bleeding events were not affected. A review of the literature showed that UAE + M TX did not significantly reduce the reintervention rate and hysterectomy rate (7.12% vs. 6.48%, P > 0.05; 1.74% vs. 1.39%, Fig. P > 0.05), it was considered that the use of MTX did not significantly improve the treatment outcome.

This study, showed that hysteroscopic uterine curettage following UACE prior to hysteroscopy and curettage for CSP has a was similar to UAE in terms of proceduresurgical success rate, intraoperative bleeding volume, and complication rate (P > 0.05). It is worth noting that the time of menstruation recovery postoperative resumption of menses than the UAE group (P < 0.05), and intraoperative blood loss was increased (43.77 ± 102.38 ml vs. 17.78 ± 30.48 ml, P > 0.05), but there was no significant difference. Intraoperative bleeding volume can be influenced by multiple factors, such as different types of CSP, gestational age, the use of MTX dose, patient response to MTX, UAE embolization endpoint judgment of the embolization endpoint, and a variation of the presence of uterine artery^12^ variations, all of which may affect intraoperative bleeding^2^,^11^,^12^. For the current study, UAE with additional MTX did not show a significant advantage for increasing the success rate and reducing bleeding events. Therefore, Mmore high-quality evidence is still needed to evaluate the clinical efficacynecessity for MTX used in of UACE versus UAE in the future. It remains controversial whether UAE treatment with CSP impacts fertility.

A meta-analysis 133 indicated of 3598 CSP patients showed that women who have had a previous CSP face an increased risk of recurrence, miscarriage, preterm birth, and placenta accreta spectrum. However, there is currently insufficient evidence to determine whether the choice of management can affect the reproductive outcomes for women who have experienced CSPthe recurrence rate of CSP was 17.6%, pregnancy with abortion, premature delivery, and placenta accreta disease was 19.1%, 10.3%, and 4.0%, respectively. And normal intrauterine pregnancy was 67.0%. In this study, the re-pregnancy outcome was similar in both groups. Among five pregnant patients, one gave birth to a healthy infant. Wang et al^14^. concluded that UAE did not induce a significant difference in pregnancy rate and live birth rate, compared with the non-UAE group. Some have argued that

In this study, 5 of 59 CSP pregnancies occurred again, including 2 recurrences and 3 intrauterine pregnancies, with a recurrence rate of 3.4% (2/59) and a normal intrauterine pregnancy rate of 60% (3/5)^14^. despite there being a possibility of occlusion for the ovary artery during It is believed that UAE, the incidence of clinically apparent injury to ovarian reserve is low^15^. They suggested that women has little effect on ovarian function in patients under 450 years of age mostly do not experience observable impact on the ovary. However, for women who are over 45 years, there is a considerably higher risk of reduction in ovarian reserve. and does not adversely affect their fertility. It has also been suggested^15^ that UAE may cause diminished ovarian function in patients over 45 years of age. Therefore, before selecting UAE treatment, the patient's condition should be fully assessed and the indication for treatment should be strictly grasped. In addition, the interventionalist should continuously improve the operation technique, select absorbable gelatin sponge during the operation, and avoid embolization of ovarian feeding arteries, etc., so as to minimize the impact of UAE on fertility.

## Conclusion

In summary, UACE was as effective and safe as UAE in the treatment of CSP either UACE or UAE combined with hysteroscopy is safe and effective in the treatment of CSP. The UACE shortened the time of menstruation recoveryto menstrual return compared withto UAE alone, and both hadwith a similar re-pregnancy fertility outcomes. However, UACE did not show a significant clinical advantage. Larger-scale clinical studies are needed to confirm the superiority of UACE in the treatment of CSPThe limited sample size of this study, which was a single-centre, retrospective study, has limitations and more evidence is needed from future multi-centre, large sample, prospective studies.
